# Clinicopathological and imaging features of struma ovarii: a retrospective study

**DOI:** 10.3389/fonc.2025.1487812

**Published:** 2025-05-15

**Authors:** Mei Chen, Shusheng Liao, Youfeng Xu, Xianwang Ye, Xiupeng Jia, Shengmin Zhang

**Affiliations:** ^1^ Department of Ultrasonography, The First Affiliated Hospital of Ningbo University, Ningbo, Zhejiang, China; ^2^ Department of Ultrasound, The First Affiliated Hospital of Wenzhou Medical University, Wenzhou, Zhejiang, China; ^3^ Department of Radiology, The First Affiliated Hospital of Ningbo University, Ningbo, Zhejiang, China; ^4^ Department of Pathology, The First Affiliated Hospital of Ningbo University, Ningbo, Zhejiang, China

**Keywords:** struma ovarii, ovarian teratoma, imaging, ultrasound, pathology

## Abstract

**Objectives:**

The struma ovarii (SO) is a rare disease, presenting significant challenges in achieving an accurate diagnosis. This study aims to delineate the clinicopathological and multimodal imaging characteristics of SO, enabling the precise diagnosis of this entity.

**Methods:**

A retrospective analysis was conducted of the clinicopathological manifestations, laboratory examination results, and imaging data (including ultrasound and MRI) of 40 SO patients who received treatment at the First Affiliated Hospital of Ningbo University over the period from January 2010 to February 2024.

**Results:**

The pathological results of 40 patients showed 21 pure SO, including 20 benign and one malignant; 19 patients had mixed SO, composed of 17 benign and two malignant. Three malignant cases were thyroid carcinoid. According to the ultrasound image, 40 SO patients were divided into three types: 22 cases were cystic, which had mainly single room or separations; 12 cases were mainly cystic-solid mixed, which had multiple septations and hyperechoic nodules; and six solid cases showed regular or irregular mass and rich blood supply. Five patients underwent contrast-enhanced ultrasound (CEUS) examination. The cystic wall and septation showed equal enhancement and slow regression. Two patients showed low enhancement in the protrusion and with slow regression. The MRI manifestations of SO are various. In this cohort, T1-weighted imaging (T1WI) showed hypo-intensity in six cases and heterogeneity in six cases; T2-weighted imaging (T2WI) showed hypo-intensity in two cases, heterogeneity in five cases, and hyper-intensity in four cases; diffusion-weighted imaging (DWI) showed hypo-intensity in two cases, heterogeneity in three cases, and hyper-intensity in four cases.

**Conclusions:**

The incidence of SO is low, but there are some features in imaging. There were cystic, cystic-solid, or solid lesions in the appendage area; CEUS showed separations and solid components with rich blood flow and slow enhancement; MRI showed high signal on T2WI and multiple cysts and heterogeneous signal on T1WI, which were the imaging markers for SO. A comprehensive understanding of imaging manifestations can help radiologists identify this disease and provide a basis for an appropriate therapeutic regime.

## Introduction

Struma ovarii (SO) is a rare highly specific mature teratoma originating from ovarian primordial germ cells. Although the thyroid tissue component is observed in 5%–15% of ovarian teratomas, the diagnosis of SO can be established when the thyroid tissue component exceeds 50% (1). SO contributes approximately 3% of ovarian teratoma, 2% of ovarian germ cell tumors, and merely 0.5% of all ovarian tumors (2). Most of the SO cases were benign, but still 0.3%–5% of them were malignant (3). SO can occur at any age, but the majority age of patients ranged from 40 to 50 years (4). The clinical manifestations of the disease, including asymptomatic pelvic mass, abdominal discomfort, thyrotoxicosis, ascites, and elevated Cancer antigen 125 (CA125), lack specificity and often resemble those of other ovarian tumors (5). The biological behavior of SO is difficult to predict, and even benign cases can transfer to malignant growth of peritoneum and omental dissemination (6), while malignant SO can develop distant metastasis (7). Overlap in the imaging manifestations with other ovarian malignancies (8) creates difficulties in the diagnosis of SO and affects the choice of surgery, especially considering the protection of the fertility of women of childbearing age (9). Therefore, early diagnosis is very important for the management and prognosis of patients. Most previous works of literature focused only on ultrasound or CT and MRI diagnosis (6, 9, 10), and there were no reports of multimodal imaging for systematic description of this disease. This study retrospectively analyzed the clinicopathological and imaging data of 40 SO patients and subdivided their ultrasound features, aiming to achieve a better understanding of imaging findings and enhance the diagnostic precision of this rare entity.

## Materials and methods

### Study subjects

This study included 40 patients diagnosed with SO at our hospital from January 2010 to February 2024. The ages of these patients ranged from 23 to 78 years, with an average age of 45 ± 15.4 years. We conducted a comprehensive retrospective analysis of the clinical data, laboratory results, and imaging findings for these patients. Our study was approved by the Medical Ethics Committee of our institution (No. 2024-041RS-01).

### Ultrasound examination

The equipment for this study included the GE E8 (Voluson E8, GE Healthcare, Chicago, IL, USA), Toshiba Aplio 500 (Tokyo, Japan), and Philips IU22 (Philips, Bothell, WA, USA) color Doppler ultrasound diagnostic systems. Transvaginal sonography and transabdominal ultrasound used a probe with a frequency range of 5–9 MHz and 3.5–5.0 MHz, respectively. The two-dimensional ultrasound evaluated the lesions’ location, size, shape, boundaries, internal echo characteristics, and the presence of pleuritic and abdominal effusion. Color Doppler flow imaging (CDFI) was utilized to analyze the blood flow within the lesions. Additionally, five patients underwent contrast-enhanced ultrasound (CEUS). This procedure involved the administration of a contrast agent (SonoVue, Bracco, Italy) in a 1.8-mL dose via the median antecubital vein. Throughout the contrast-enhanced phase, continuous observation was made of the enhancement pattern, timing, intensity, and washout of the contrast agent within the lesions.

### MRI inspection method

MRI examinations were conducted using a GE Signa HDxt 1.5T superconducting MRI scanner. A phased array body coil was utilized with the following scan parameters: for sagittal T2-weighted imaging (T2WI) with fat suppression, the repetition time (TR) was 5,300 ms, and the echo time (TE) was 102 ms; for T1-weighted imaging (T1WI), the TR was set to 600 ms and the TE to 7 ms; T2WI with lipid suppression had a TR of 4,900 ms and a TE of 108 ms; T2WI in the coronal position featured a TR of 4,940 ms and a TE of 102 ms; T1WI with fat suppression in the axial plane had a TR of 760 ms and a TE of 3.6 ms; slice thickness ranged from 5.6 to 6.0 mm, with a slice gap of 2 mm. For enhanced scanning, a transverse and sagittal T1WI sequence with lipid suppression was used, employing gadolinium gluconate (Gd-DTPA) as the contrast agent at a dose of 0.2 mmol/kg and an injection rate of 2–3 mL/s. The parameters for this phase were adjusted accordingly. Diffusion-weighted imaging (DWI) was performed in the axial plane using a single-shot echo-planar imaging sequence with a TR of 5,125 ms, a TE of 98 ms, b-values of 0 and 1,000 s/mm^2^, a slice thickness of 6 mm, an interslice gap of 2 mm, and an acquisition number of 4.

### Statistical method

Statistical analyses were conducted utilizing SPSS version 23.0. Continuous variables are reported as either mean or median values, measurement data are expressed as $\bar{x}$ ± s, and categorical variables are described using frequencies and percentages.

## Results

### General medical history

Among the 40 patients, 25 (62.5%) patients were premenopausal, and 15 (37.5%) were postmenopausal. Of the patients, 28 (70.0%) were asymptomatic, and 12 (40.0%) had different symptoms, such as abdominal swelling, vaginal bleeding, pelvic pain, or digestive symptoms. Of the cases, 15 (37.5%) had minimal ascites, three cases (7.5%) had moderate ascites, and 17 (42.5%) had no ascites. There were thyroid nodules in five patients (12.5%) who had undergone scans of their thyroid. In this cohort, 20 cases performed laparoscopic ovarian cyst removal, 10 cases underwent salpingo-oophorectomy laparoscopy, three cases underwent laparoscopic salpingectomy, and seven cases underwent total transabdominal adnexectomy. Compared to menstruation, the symptomatology, ascites, surgery, and pathology between the pure and impure groups had no statistically significant differences (**Table 1**).

**Table 1:** Clinical characteristics and surgical management of the 40 patients with SO.

Parameters		Pure (n = 21)	Impure (n = 19)
Age at surgery (years)		44 (29–71)	45 (23-78)
Menstruation	Premenopausal	30.0% (12/40)	32.50% (13/40)
	Postmenopausal	22.5% (9/40)	10.0% (4/40)
Symptomatology	Asymptomatic	37.5% (15/40)	32.5% (13/40)
	symptomatic	15.0% (6/40)	15.0% (6/40)
Ascites	Mild	17.50% (7/40)	20.0% (8/40)
	Moderate	7.50% (3/40)	0
	Severe	0	0
	No	25.0% (10/40)	17.50% (7/40)
Surgery	Laparoscopic oophorocystectomy	25.0% (10/40)	25.0% (10/40)
	Salpingo-oophorectomy laparoscopy	12.50% (5/40)	12.50% (5/40)
	Total laparoscopic hysterectomy	2.50% (1/40)	5.0% (2/40)
	Total abdominal hysterectomy	12.50% (5/40)	5.0% (2/40)
Pathology	Benign	50.0% (20/40)	42.50% (17/40)
	Malignant	2.50% (1/40)	5.0% (2/40)

Values are expressed as median (range) or n (%).

SO, struma ovarii.

### Laboratory test results

1) Tumor markers: The level of CA125 was higher than 118.9 U/mL (normal, <30 U/mL) in four cases (10.8%). Two cases had higher human chorionic gonadotropin (HCG) levels for pregnancy, and one had also a higher serum alpha-fetoprotein (AFP) of 162 U/mL (normal, <5 U/mL). The level of Cancer antigen 724 (CA724) in two cases was slightly high at 10.89 U/mL (normal, <7 U/mL). In three cases, Cancer antigen 199 (CA-199) was slightly high at 31.3 U/mL (normal, <30 U/mL). One case had a high serum carcinoembryonic antigen (CEA) at 6.4 ng/mL (normal, <5 ng/mL). 2) Serum sex hormones: Five cases had high E2, one case had high prolactin (PRL), and four cases had high TT. 3) Serum thyroid hormone: The thyroid indicators in most patients were in the normal range (**Table 2**).

**Table 2:** Laboratory results of 40 patients.

Parameters			Pure (n = 21)	Impure (n = 19)
Serum tumor marker	Serum CA125 level (U/mL) Reference range (0–30)	<30	45.95% (17/37)	43.24% (16/37)
		>30	5.41% (2/37)	5.41% (2/37)
	Serum CA-724 level (U/mL) Reference range (0–7)	<7	48.48% (16/33)	45.45% (15/33)
		>7	3.03% (1/33)	3.03% (1/33)
	Alpha-fetoprotein (AFP) level (ng/mL) Reference range (0–25)	<25	50.0% (18/36)	47.22% (17/36)
		>25		2.78% (1/36)
	Carcinoembryonic antigen (CEA) Level (ng/mL) Reference range (0–5)	<5	41.94% (13/31)	54.84% (17/31)
		>5		3.23% (1/31)
	Serum CA-199 level (U/mL) Reference range (0–25)	<37	42.42% (14/33)	48.48% (16/33)
		>37	6.06% (2/33)	3.03% (1/33)
Serum sex hormone	Estrogen levels (E2) (IU/L)	Normal	46.67% (14/30)	36.67% (11/30)
		Abnormal	6.67% (2/30)	10.0% (3/30)
	Prolactin (PRL)	Normal	56.61% (16/31)	45.16% (14/31)
		Abnormal	3.23% (1/31)	
	Testosterone (TT)	Normal	42.86% (12/28)	42.86% (12/28)
		Abnormal	3.57% (1/28)	10.71% (3/28)
Serum thyroid hormone levels and antibodies	Triiodothyronine (T3)	Normal	43.33% (13/30)	46.67% (14/30)
		Abnormal	3.33% (1/30)	6.67% (2/30)
	Thyroxine (T4)	Normal	44.83% (13/29)	48.28% (14/29)
		Abnormal		6.70% (2/29)
	Thyroid-stimulating hormone (TSH)	Normal	39.29% (11/28)	53.57% (15/28)
		Abnormal	7.14% (2/28)	
	Thyroid peroxidase antibody (TPO)	Normal	44.44% (4/9)	33.33% (3/9)
		Abnormal	22.22% (2/9)	

### Pathological and immunohistochemical examination results

**Figure 1:**
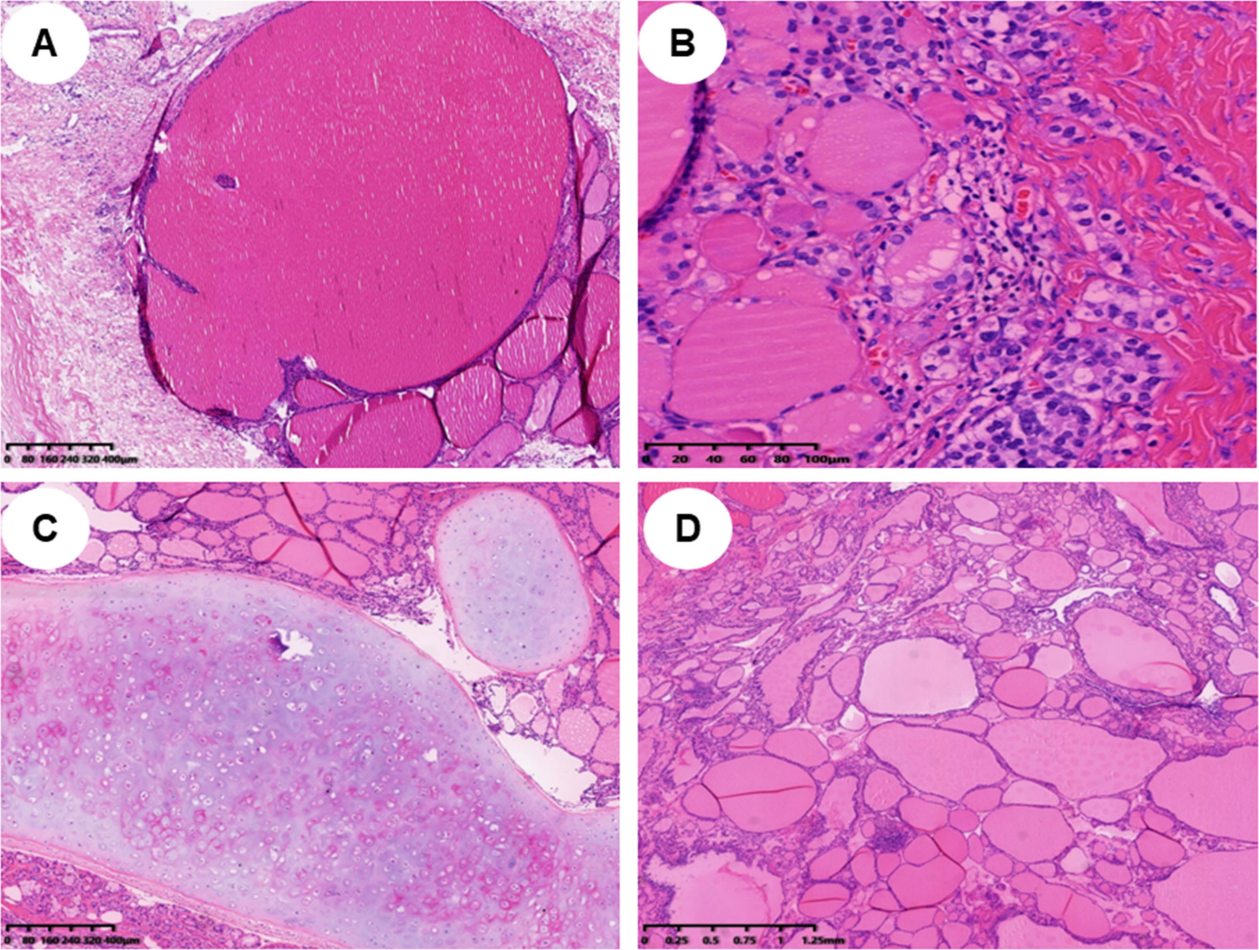
Histopathological findings of different types of struma ovarii (H&E staining). **(A)** Histopathological findings of a pure struma ovarii (×40). **(B)** Histopathological findings of a struma carcinoid (×200). **(C, D)** Histopathological findings of an impure struma ovarii; mature teratoma component can be found in panel C (×40), and struma ovarii tissue is displayed in panel D (×40).

**Figure 2:**
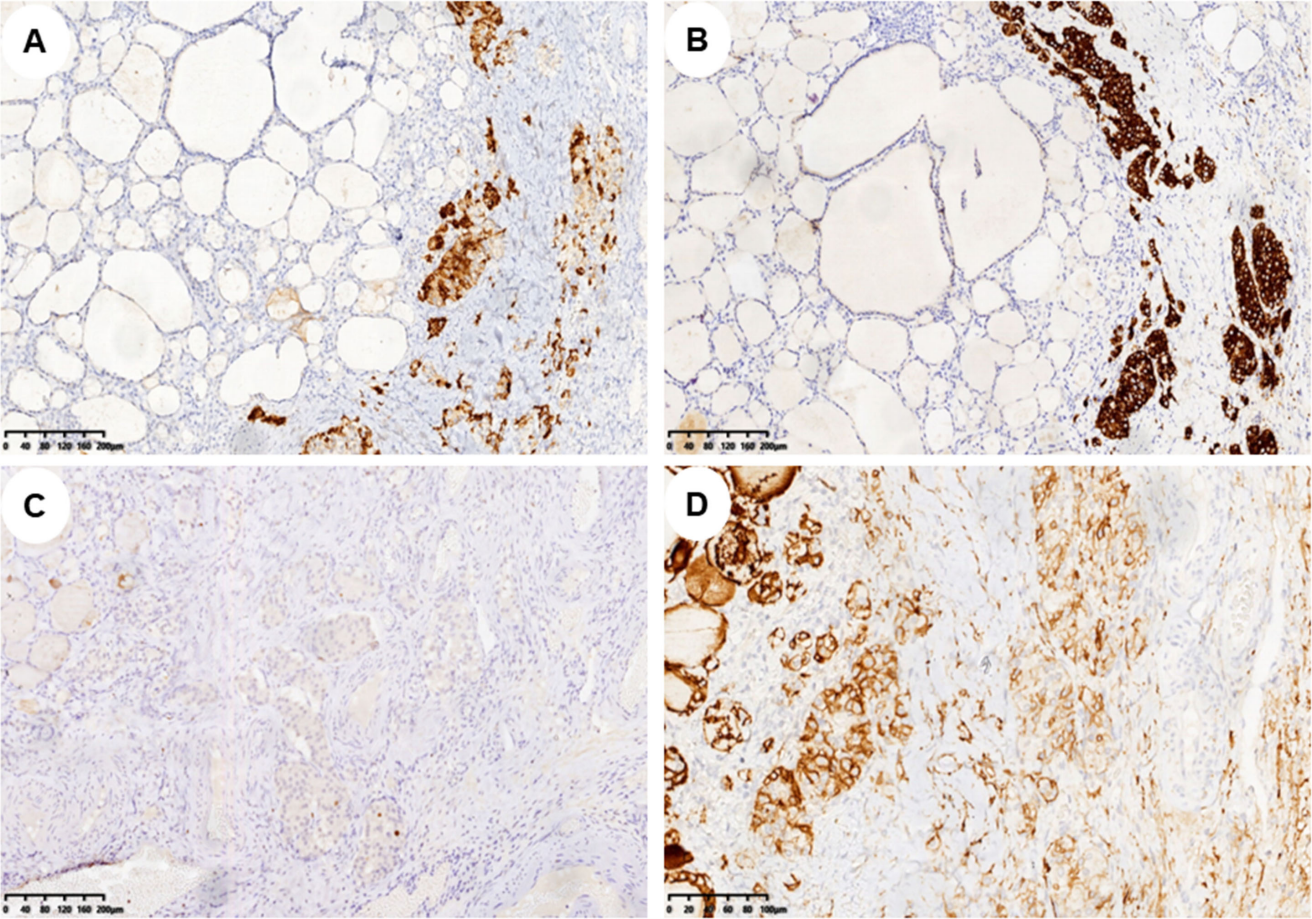
Immunohistochemical markers of struma carcinoid. **(A–D)** Immunohistochemistry showed CgA, Syn, Ki-67, and CD56 expression in struma carcinoid cells, respectively (**A–C**, ×100; **D**, ×200). CgA, chromogranin A; Syn, synaptophysin; CD56, cytokeratin 5/6.

In pathology, 21 (52.5%) patients had pure SO (**Figure 1A**), and 19 (47.5%) patients had impure SO (**Figures 1C, D**). The majority of 37 (92.5%) patients were benign, only three (7%) patients were malignant, and the pathological subtypes of malignant were strumal carcinoid (**Figure 1B**). In these malignant cases, one had pure SO, and the other two had impure SO. The postoperative follow-up of three malignant patients was 8, 20, and 141 months, and there was no metastasis or death. The 37 benign patients had no recurrence during the follow-up period. The immunohistochemical examination was further carried out for three malignant patients, and the expression of tumor cell immune markers across them is detailed in **Supplementary Table**. Notably, there was a high expression of chromogranin A (CgA), synaptophysin (Syn), and cytokeratin 5/6 (CD56) in all three malignant patients and thyroglobulin (TG) in two patients, with Ki-67 between 1% and 2% (**Figure 2**). In contrast, the results revealed no expression of calcitonin in all three patients and TG in two patients.

### Ultrasound features of SO

#### 2D and color Doppler ultrasound manifestations of 40 SO patients

**Figure 3:**
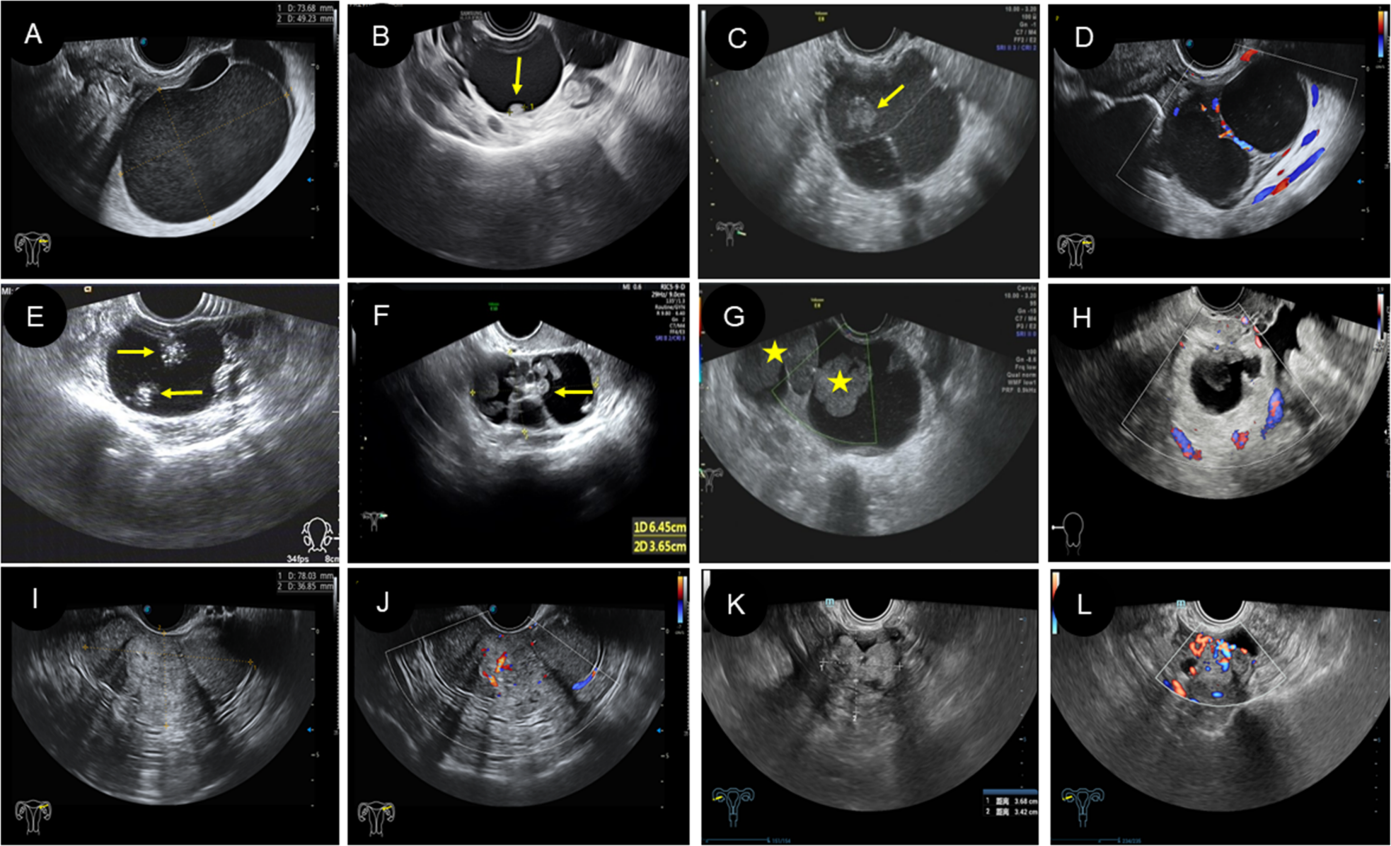
Two-dimensional and color Doppler ultrasound manifestations of different types of struma ovarii. **(A–D)** The ultrasound manifestations of cystic type of SO. **(A)** Two-dimensional ultrasound showed a large cyst with regular morphology and clear boundary, and the cyst is filled with weak echoes. **(B)** Two-dimensional ultrasound revealed a unilocular cyst with a papillary protrusion (arrow) in the cystic wall. **(C)** Two-dimensional ultrasound revealed multilocular cyst with several septations and “amniotic fat ball” (arrow) within the cyst. **(D)** On color Doppler flow imaging, blood flow signals were visible in the cystic wall and septa. **(E–H)** The ultrasound features of cystic-solid-type SO. **(E, F)** Two-dimensional ultrasound showed cystic-solid SO with several hyperechoic and different-sized nodules (arrow) in the cystic area (known as “strum pearls”). In addition, thick septa can be found in panel **(F, G)** The “amniotic fat ball” (asterisk) also can be found in the cystic area of cystic-solid SO patients. **(H)** On CDFI, blood flow signals were visible in the solid component. **(I–L)** The ultrasound characteristics of solid type of SO. **(I, J)** A solid-type SO patient showed regular morphology with rich blood flow. **(K, L)** A solid-type SO patient showed irregular morphology with rich blood flow. SO, struma ovarii; CDFI, color Doppler flow imaging.

1) Lesion site: All patients were unilateral, including 25 patients on the left side and 15 patients on the right side. 2) Size: The diameter of the mass ranged from 2.6 to 16.2 cm. 3) Morphology and boundary: All patients had clear boundaries. Of the cases, 31 (77.5%) were round or circular, and the morphology was regular. Nine cases (22.5%) had irregular morphology. 4) Internal echo manifestations and blood flow: According to ultrasound features, the patients were subdivided into three types: 22 (55.0%) were cystic type, 12 (30.0%) were cystic-solid type, and six (15.0%) were solid type. Among the 22 cystic cases, single room and thick septations were the features (**Figures 3A, D**). There were eight cases that showed papillary protrusion on the cystic wall (**Figure 3B**) and an “amniotic fat ball” floating inside the cyst (**Figure 3C**), and some had a “catkins” sign hanging on the wall. Twelve cases of cystic-solid (**Figure 3G**), light spots, and separations were seen in most cases. In particular, there were “strum pearls” (**Figures 3E, F**) in seven cases, which were seen as hyperechoic nodules with different sizes and rich blood flow within them (**Figure 3H**). Among the six solid ones, there were regular or irregular nodules with clear boundaries (**Figures 3I, K**), which demonstrated rich blood flow in both peripheral and internal areas (**Figures 3J, L**), with moderate resistance index (RI). Among this cohort, only two cases had concurrent presence of myoma or polyp. According to the International Ovarian Tumor Analysis (IOTA) system classification, 14 (35%) cases were diagnosed to be benign, 13 (32.5%) cases were borderline, and 13 (32.5%) cases were malignant (**Table 3**).

**Table 3:** Two-dimensional and color Doppler ultrasound findings of the 40 patients of SO.

Ultrasonic classification		Cyst (n = 22)	Cystic-solid (n = 12)	Solid (n = 6)
Number		55.0% (22/40)	30.0% (12/40)	15.0% (6/40)
Position	Left	32.50% (13/40)	22.50% (9/40)	7.50% (3/40)
	Right	22.50% (9/40)	7.50% (3/40)	7.50% (3/40)
Maximum diameter (cm)		2.7–12.5	2.6–16.2	3.7–7.8
Shape	Regular	50.0% (20/40)	17.5% (7/40)	10.0% (4/40)
	Irregular	5.0% (2/40)	12.5% (5/40)	5.0% (2/40)
Cystic fluid	Clear	15.0% (6/40)	11.76% (4/40)	
	Unclear	40.0% (16/40)	20.0% (8/40)	
	No			15.0% (6/40)
Separation	Yes	25.0% (10/40)	20.0% (8/40)	
	No	30.0% (12/40)	10.0% (4/40)	15.0% (6/40)
Parenchymal echo and blood flow in the sac	Yes	20.0% (8/40)	17.5% (7/40)	0
	No	35.0% (14/40)	12.5% (5/40)	15.0% (6/40)
Comet tail sign	Yes	15.0% (6/40)	22.50% (9/40)	
	No	40.0% (16/40)	7.5% (3/40)	15.0% (6/40)
Ultrasound diagnosis (IOTA system)	Benign	30.0% (12/40)	12.5% (5/40)	
	Uncertain	15.0% (6/40)	10.0% (4/40)	
	Malignant	10.0% (4/40)	7.50% (3/40)	15.0% (6/40)

Values are expressed as n (%).

SO, struma ovarii.

### CEUS features of SO

**Figure 4:**
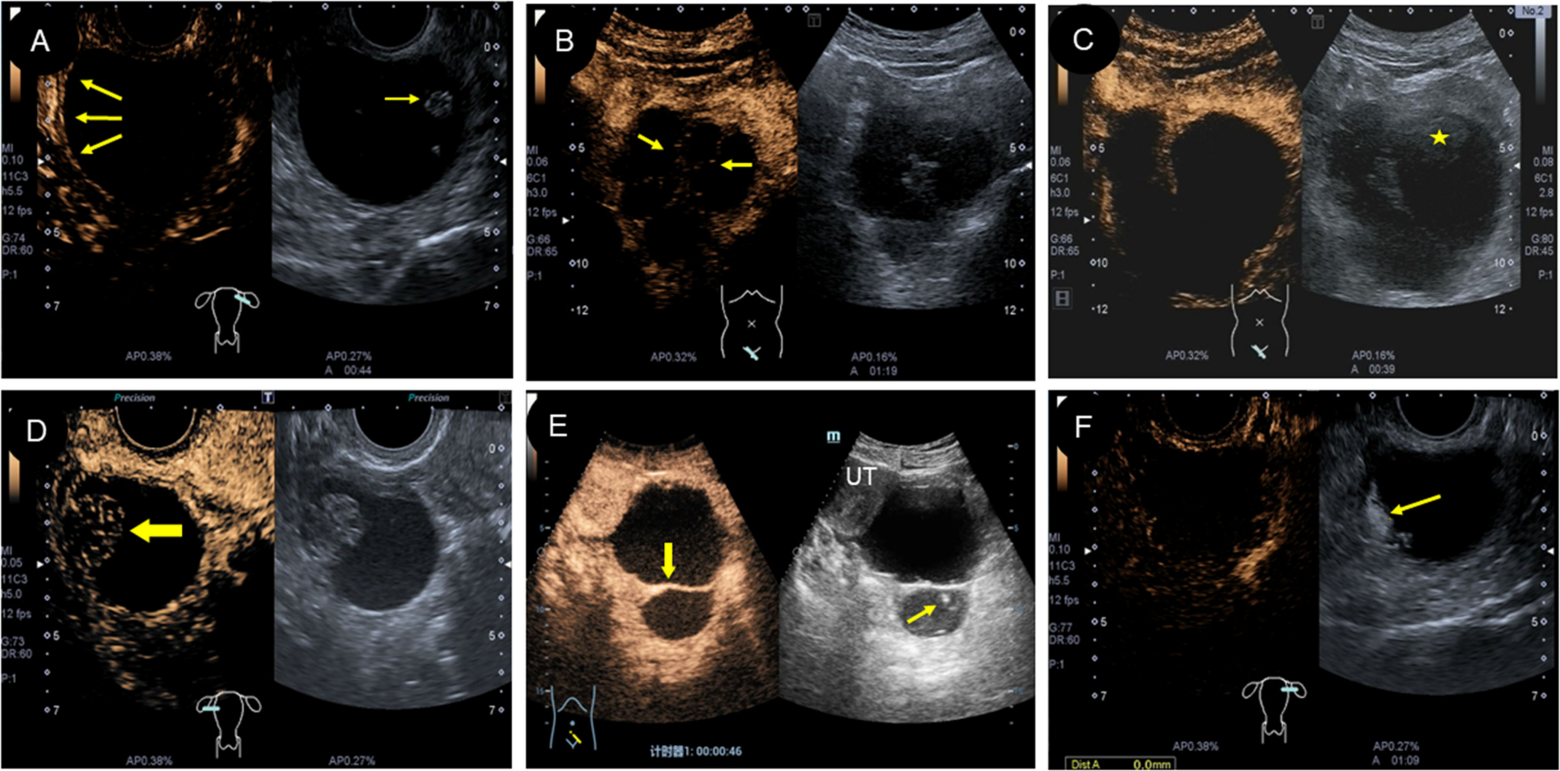
Characteristics of CEUS in different types of struma ovarii. **(A)** CEUS showed low enhancement of the cyst wall and papillary protrusion (arrow). **(B)** and **(E)** Different forms of septations in cyst were enhanced, but the light spot was unenhanced. **(C)** The “amniotic fat ball” (asterisk) in cyst showed no contrast agent perfusion on CEUS. **(D)** CEUS displayed that the solid area (arrow) was low enhanced in a cystic-solid-type SO. **(F)** CEUS showed that the irregular protrusion in cyst wall (arrow) was low enhanced, which was verified as a struma carcinoid pathologically. CEUS, contrast-enhanced ultrasound; SO, struma ovarii.

Five patients in this group underwent a CEUS examination. Among them, four cases were cystic with papillary protrusion and separations. After injecting the contrast agent, the cystic wall and septations were enhanced, ranging from 13 to 19 s and showing equal enhancement (compared to the degree of uterine enhancement) and subsequently with slow regression (**Figures 4A, B, E**). In two cases, contrast agent perfusion was visible at the intracapsular protrusion (**Figures 4D, F**), which showed slow enhancement and then slow regression, and the other two cases showed no contrast perfusion at the intracapsular protrusion. In one case of cystic-solid type, contrast perfusion was seen in the cyst wall and septa after injection of contrast agent; however, there was no contrast perfusion in the heterogeneous moderate echo components (“amniotic fat ball”) within the capsule (**Figure 4C**).

### The MRI performance

**Figure 5:**
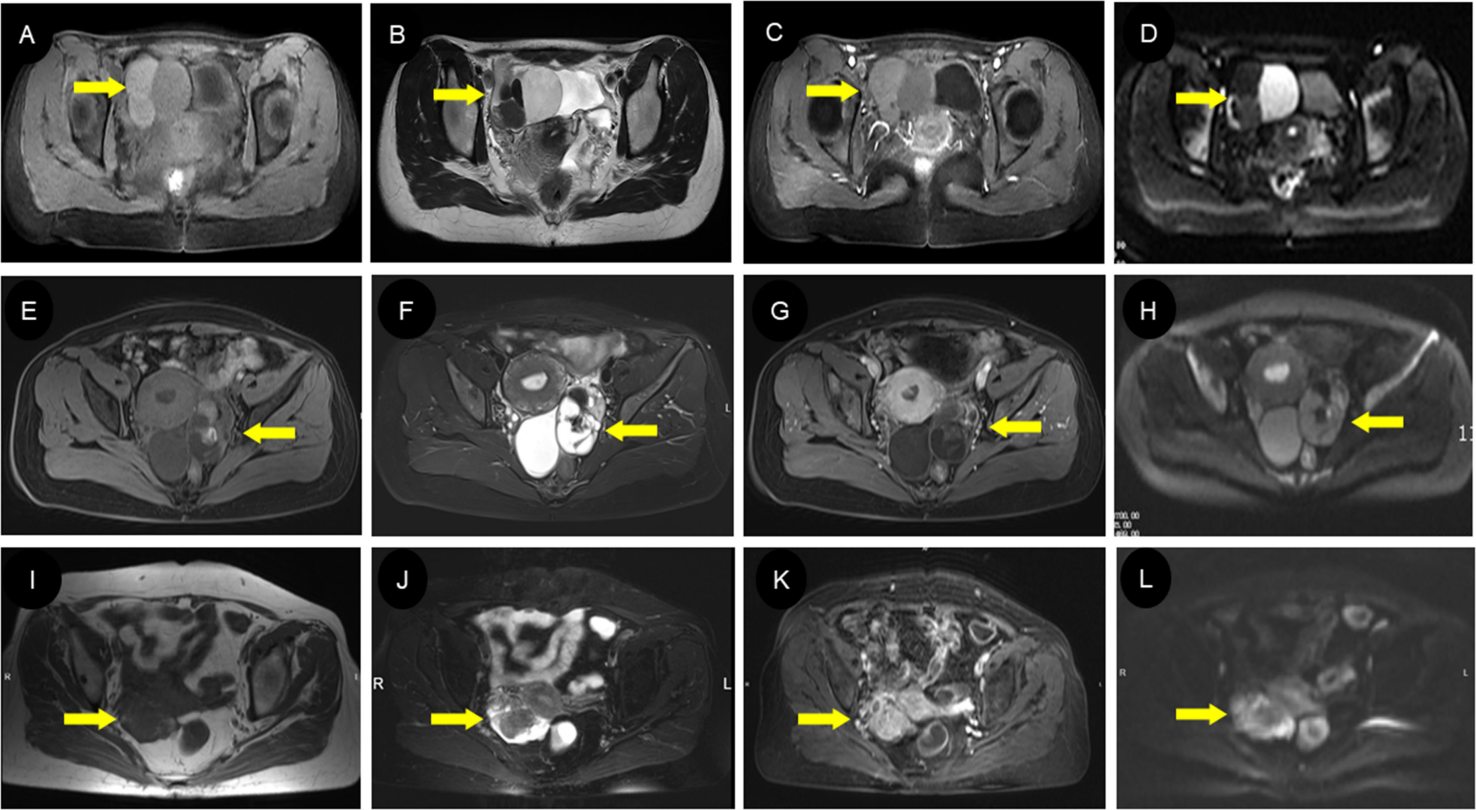
MRI findings of different types of struma ovarii. **(A–D)** A 23-year-old woman with cystic-type SO with multiple circular signal shadows in the right ovary. The cyst had slightly low signal in axial T1WI **(A)** and high/low signal in T2WI **(B)** with enhanced internal septa **(C)**. The DWI images **(D)** exhibited diverse cyst signals, suggestive of the varying viscosity of the cyst fluid, which can be indicative of a diagnosis of SO. The extremely low signal on T2WI **(B)** represents the colloidal substances consisting of thyroglobulin, thyroid hormones, and so on, which is of great value in diagnosing SO. **(E–H)** A 44-year-old woman with mixed-type SO with a solid and cystic mass in the left ovary. There was heterogeneous signal intensity on both T1WI **(E)** and T2WI **(F)** owing to regions of fluid, hemorrhage, and calcification; prominent contrast enhancement was seen in the solid part **(G)**. Locally hyperintense regions on the DWI **(H)** are presented on the DWI images, suggesting a higher concentration of thyroglobulin within the lesion. **(I–L)** A 71-year-old woman with solid-type SO with a heterogeneous lesion in the right ovary. Most parts of the lesion showed iso-intensity on both T1WI **(I)** and T2WI **(J)** with vivid uneven enhancement **(K)**, while the surrounding wreath-like cystic part presented slightly hyperintense signals on T1WI **(I)**, markedly hyperintense signals on T2WI **(J)**, and hyperintense signals on DWI **(L)**. SO, struma ovarii; T1WI, T1-weighted imaging; T2WI, T2-weighted imaging; DWI, diffusion-weighted imaging.

Twelve patients in this study underwent an MRI examination. T1WI showed hypo-intensity in six cases and heterogeneity in six cases (**Figures 5A, E, I**). T2WI showed hypo-intensity in two cases, heterogeneity in five cases, and hyper-intensity in four cases (**Figures 5B, F, J**). DWI showed hypo-intensity in two cases, heterogeneity in three cases, and hyper-intensity in four cases (**Figures 5D, H, L**). All the 10 cases with enhanced scans showed obvious enhancement (**Figures 5C, G, K**). Half of these 12 patients were finally confirmed to have simple SO by pathology (**Table 4**).

**Table 4:** Magnetic resonance imaging (MRI) features of the 12 patients of SO.

MRI Project	Pure SO (n = 6)			Impure SO (n = 6)		
	Hypo-intensity	Heterogeneity	Hyperintensity	Hypo-intensity	Heterogeneity	Hyperintensity
T1WI	2 (16.67%)	4 (33.33%)		4 (33.33%)	2 (16.67%)	
T2WI		3 (25.0%)	2 (16.67%)	2 (16.67%)	2 (16.67%)	2 (16.67%)
DWI	2 (16.67%)		3 (25.0%)		3 (25.0%)	1 (8.33%)

Values are expressed as n (%).

SO, struma ovarii; T1WI, T1-weighted imaging; T2WI, T2-weighted imaging; DWI, diffusion-weighted imaging.

## Discussion

SO was defined by the WHO in the early 20th century as a monodermal teratoma, which was composed mainly or only of the thyroid tissue (1). Previous studies have shown that SO can occur at any age (7), but mostly during menopause. A few studies have also reported that SO is visible during pregnancy (11), which may be related to the transient increase of thyroid cancer risk in this situation (12). This was mainly due to the structural similarity between HCG and thyroid hormone. HCG can stimulate the thyroid-stimulating hormone (TSH) receptor in ovarian tumors and promote tumor growth during pregnancy (13). Two cases in this study also occurred during pregnancy, and tumor resection was performed during a cesarean section. The majority of SO patients had no special clinical symptoms; previous studies have reported that abdominal pain was the main clinical symptom, and it was believed that the symptoms of pain were caused by increased pressure in the pelvis (5). In this study, 12 (30.0%) patients had the clinical symptoms of abdominal pain and distension, which slightly increased, compared with the previous studies. A few patients with SO may have pseudo-Meigs syndrome, which can disappear after tumor resection (4, 14). Peyron (15) suggested that the increased CA125 may be related to the stimulation of the peritoneal, omentum, and mesentery mesothelial cells. In this study, the variable occurrence of ascites was found in 18 (45%) patients, and abdominal effusion disappeared after the lesions were resected.

Pathologically, benign SO is the most common. However, there have approximately 5% of cases of malignant SO (3). In this study, 37 (92.5%) patients were benign, and only three patients were malignant, which coincided with previous studies (3, 16–18). Carcinoid tumors are similar to well-differentiated neuroendocrine tumors in other organs (19), usually asymptomatic, and a small number present with constipation, abdominal distension, and pain, which are due to the production of YY peptides (20). In malignant tumors, metastasis and recurrence are positively correlated with the Ki-67 proliferation index and tumor volume (19–22). In our study, the three malignant patients were asymptomatic with a long history and classified at the stage of International Federation of Gynecology and Obstetrics (FIGO) I. Two of them had a Ki-67 index of less than 2%, and the average tumor diameter was small at approximately 0.3 cm. No metastasis occurred during follow-up, which is consistent with previous studies (23, 24).

Immunohistochemistry is an important basis for the diagnosis and differential diagnosis of tumors. In our study, all three patients with thyroid carcinoid were positive for CgA, Syn, and CD56, which is consistent with the previous literature (25, 26). All of them were excluded from the diagnosis of medullary thyroid carcinoma due to negative calcitonin staining. One of them was diagnosed with a follicular variant of papillary thyroid carcinoma due to positive CD56 and negative HBME1. Another one was confirmed as mucinous cystadenoma because of positive TTF-1, which was marked with epithelial-origin gynecological tumors (26).

Regarding tumor markers, the levels of CA125, CA724, and CA-199 were slightly elevated in some patients, which is consistent with a previous study (4). In this study, the levels of sex hormones and thyroid hormones, such as E2, TT, and free triiodothyronine (T3), were elevated in some patients. It is speculated that as a neuroendocrine tumor, carcinoids can cause abnormal levels of estrogen and thyroid hormones (27). However, T3 and thyroxine (T4) were elevated in some cases with a normal TSH, which may be ascribed to thyroid tissue in SO. Some literature reported that 5%–8% of SO may be accompanied by hyperthyroidism (1). Therefore, if a female patient has abnormal thyroid function, the ovary should be routinely examined to rule out SO.

SO ultrasonic manifestations were varied. In this study, we classified SO patients into cystic, cystic-solid, and solid types. Among them, the cystic type was the most common, accounting for approximately 55%. Most of them can be seen with thick walls and separations, also with “comet tail” signs of glial deposition. The formation mechanism of the comet tail sign may be similar to that of a thyroid glial cyst (25). Solid-cystic type was often larger, which may be related to the secretory function of thyroid tissue, and the continuous secretion of glial cells leads to the enlargement of tumor mass (25). More internal compartments and nodular or papillary protrusions were seen in the capsule wall and compartments. This nodular or papillary protrusion was also called “thyroid pearl” (28), which was a special ultrasonic manifestation. The size of the “thyroid pearl” was generally small, and it could be single or multiple, and CDFI showed rich blood flow within it. However, some components were similar to “amniotic fat ball”, which were floating in the sac; CDFI did not show obvious blood flow, and most of them were colloids secreted by thyroid tissue. More interesting, most of the capsule walls had a “comet tail” too. The solid type was characterized by a well-defined boundary and moderate internal echo, and CDFI showed rich blood flow. This type overlapped with many other ovarian malignancies. According to the IOTA system (29), cystic with multiple septa, solid-cystic, and solid types of SO could be easily misdiagnosed. In this study, all the cases had clearly boundaries; even 26 cases were diagnosed as malignant or unclear type by ultrasound, but only three cases were finally malignant. A review of the ultrasound images revealed that the tumor had clear boundaries and a moderate internal echo similar to thyroid tissue, which may be the difference between SO and other ovarian malignancies (25). The rich blood flow in tumors was related to the abundant fibrovascular matrix (30). The ultrasonographic manifestations of solid-cystic and solid SO are complex and often misdiagnosed, which leads to unnecessary laparotomy and extensive operation.

The advantage of CEUS was that it could show low-velocity blood flow (31), which had been widely used. In this study, two patients of cystic type had “thyroid pearls” visible on the cyst wall, and color Doppler showed no obvious blood flow signal. However, contrast perfusion was seen by CEUS with slow and equal enhancement. The “thyroid pearl” was considered to be composed of thyroid tissue and matrix with rich blood vessels and fibers (30), which had endocrine functions. It can be found that the display of microcirculation by CEUS was superior to CDFI. In the other case, the cystic-solid type showed an “amniotic fat ball” sign on 2D ultrasound, and no contrast perfusion was observed even when using CEUS. This was because these “amniotic fat balls” were actually colloids secreted by thyroid tissue, concentrated in cysts, and without vessels inside. They looked like “thyroid pearls”. Therefore, CEUS can obtain more imaging information and help to deeply understand the pathological features of this lesion. At the same time, the visualization of intra-tumoral microcirculation by CEUS can improve the identification of benign and malignant (32). Some scholars have found the discrepancy in microcirculation density in benign and malignant tumors (33) and revealed that the solid part of ovarian cancer was characterized by high enhancement with rapid perfusion and fast regression (32, 34). In contrast, the ovarian physiological luteal hematoma showed circular enhancement on the thick wall and without contrast perfusion in the internal area (34). Benign ovarian sex cord stromal tumors showed sparse or no enhancement, while malignant tumors showed rapid and high enhancement (35). The results of this study show that SO is characterized by slow enhancement and regression on CEUS, which is more consistent with the manifestations of benign tumors.

The MRI manifestations of SO were complex. Multiple cysts and heterogeneous signals usually show low signals in T1WI. However, they change with the increase of mature fat components. In this cohort, half of the patients showed hypo-intensity in T1WI, but the other half showed heterogeneity for containing mature fat components. T2WI showed a high signal and/or very low signal, depending on the protein content of SO. The signal can gradually decrease with the increase of the viscosity in the lesions (8, 9). If there was jelly-like material in the capsule, T2WI could show a very low signal, which was the characteristic MRI appearance of SO (36). Moreover, hemorrhage in the cyst can increase the signal in the cyst (8). In this study, T2WI showed almost all cases of heterogeneity and hyperintensity for less content of jelly-like material. Diffusion-weighted imaging was capable of reflecting the diffusion patterns of water molecules within living tissue. Where the protein content and viscosity within the lesions were minimal, DWI typically demonstrated unrestricted diffusion as in previous studies (8, 37). In this study, there was no enhancement observed in the cystic portion of the tumor. However, the cystic wall, septa, and “struma pearls” exhibited prominent enhancement for abundant blood vessels and fibers. Consistently, all cases in our study exhibited marked enhancement. The presence of imaging findings that were indicative of multiple signal patterns on T1WI and T2WI reflecting the content of thyroid colloids, hypervascularity, and restricted diffusion within the solid component on DWI collectively suggest the presence of a specific condition. These comprehensive imaging features contributed to a more accurate diagnosis and understanding of the pathophysiology of SO.

### Study limitations

Several limitations of this study should be noted. First, because it is a retrospective study, some clinical and imaging data are incomplete, and only 12 patients underwent MRI examination in our study, so the contrastive analysis of different imaging in all patients is unavailable. Second, this research was derived from a single center with a relatively small sample size, so a multicenter, large sample, prospective study is needed to further increase the understanding of this rare entity.

## Conclusion

SO is a clinically rare disease and may be easily misdiagnosed. However, there are some characteristic manifestations in imageology. In cystic or cystic-solid lesions with clear boundary located in the adnexa, 2D ultrasound presents “thyroid pearl”, “amniotic fat ball”, and “comet tail” in the cystic area; CEUS shows blood flow in papillary protrusion or septation. MRI shows a high signal or confounding signal in T2WI and a low signal in T1WI but with enhanced nodules. An accurate understanding of the imaging manifestations of SO can help radiologists identify this disease and provide a basis for an appropriate clinical therapeutic regime.

## Data Availability

The raw data supporting the conclusions of this article will be made available by the authors, without undue reservation.
