# Supplementary material for: Clinicopathological and imaging features of struma ovarii: a retrospective study
Source: Front Oncol. 2025 May 15;15:1487812. doi: 10.3389/fonc.2025.1487812 (PMC12119309; doi:10.3389/fonc.2025.1487812)
Supplement: Supplementary file 1 [file Table1.docx]

Supplementary table Tumor cell immune marker expression in the 3 malignant patients

| Immunohistochemical index | Calcitonin | CgA | Syn | CD56 | TG | TTF-1 | Ki-67 |
| --- | --- | --- | --- | --- | --- | --- | --- |
| Case1 | - | + | + | + | - | NA | NA |
| Case2 | - | + | + | + | - | - | +1% |
| Case3 | - | + | + | + | + | + | <+2% |

CgA :chromogranin-A, Syn: synaptophysin, CD56:cytokeratin56, TG: thyroglobulin, NA: not available.
